# How can we improve our understanding of skillful motor control and apraxia? Insights from theories of “affordances”

**DOI:** 10.3389/fnhum.2014.00612

**Published:** 2014-08-08

**Authors:** J. C. Mizelle, Lewis A. Wheaton

**Affiliations:** ^1^Atlanta Veterans Affairs Medical CenterDecatur, GA, USA; ^2^School of Applied Physiology, Georgia TechAtlanta GA, USA

**Keywords:** apraxia, affordances, motor control, tool use, tool use behavior

A recent opinion article published in Frontiers in Human Neuroscience (Osiurak, 2013) points out several challenges of the study of “affordances” related to investigations of apraxia. In 2010, we published in Frontiers a review and theoretical proposal that addresses our concerns about affordances and grounded cognition (Mizelle and Wheaton, 2010b). Central to our premise was the argument that, for tool use, action goals were the grounded invariant elements as opposed to the action representations of the tools themselves. Further, parameters of the behavior(s) undertaken to achieve the action goal (tool used to accomplish the task, usage context of the tool, and the motor variables to accomplish the task) are affording to the goal, inherently variable, but are driven by the fixed action goal itself. Our model (Modular Selection for Action Goals, MSAG) incorporates the idea that stored representational knowledge of tools can be broadly adapted by usage context so that action goals can be achieved, emphasizing the adaptability of tool contextual and usage representations and the fixedness of the overall action goal. In commentary to our MSAG model, Pellicano et al. (2011) considered an alternative view where affordances (stable and variable) function to align tools to action goals. The core difference is that Pellicano and colleagues proposed that potentiation of tool-action goal alignment is mediated by affordances (certain properties of tools). We proposed that the fulfillment of the action goal defines affording properties of any possible combination of tools and motor variables, where some combinations are more or less affording to the action goal than others. At any rate, we certainly agree that further studies need to be considered to appreciate and refine specifics of any models, whether ours or those of Pellicano and colleagues.

While Osiurak emphasized the alternative commentary (Pellicano et al., 2011), we feel it is worth noting that many of the ideas presented by Osiurak (2013) reflect core concepts of our 2010 MSAG theory. For example, in Figure 2 of the MSAG proposal paper (Mizelle and Wheaton, 2010b), selection of alternative tools when the canonical tool is not available (we use the example of tools within a reasonable workspace) is not necessarily driven by a broad range of stable affordances (the adaptive grounded view). Rather, an alternative tool is selected based on the properties which best allow for the accomplishment of the action goal based on known mechanical/functional properties of tools. This embodies the first two assumptions of Osiurak (2013). Under MSAG, interconnected modules are triggered by an action goal that afford semantic flexibility of tools; tool (selection), usage context (refinement, as tools have multiple uses), and neurobiomechanics (motor specifics).

Further, our contention has been that the elements that best fulfill the action goal become the relevant affordances for tool selection and motor performance, not necessarily the “grounded” or stable affording properties of tools. This embodies the third assumption presented by Osiurak. The action goal defines the cooperatively determined usage context of the tool. Chiefly, this allows for creativity and adaptability in how action goals are accomplished, especially when canonical tools—those with grounded action representations coincident with the action goal—are not available.

Work in our lab has sought to understand how people “connect” tools and objects for action goals. Using electroencephalography (EEG), we have suggested that erroneous tool-object pairings generates ventral activation, which seems to precede parietofrontal activation typical of tool-object encoding for action (Mizelle and Wheaton, 2010a,c). Further, in a multimodal neuroimaging study, we used EEG and functional MRI (fMRI) to propose that contextual understanding of incorrect/impossible actions (via ventral pathways) precedes the activity of correct/possible actions (via parietofrontal pathways) that may suggest how both conceptual and ideomotor type apraxias could occur (Mizelle and Wheaton, 2010d). Indeed, this was reflected in MSAG as we proposed that ventral damage could corrupt the ability to align a tool with the action goal and the canonical usage context of that tool. In this case, a failure to *deselect* inappropriate tools would result, but the inappropriate tool would be used in motor-relevant ways in an attempt to achieve the desired behavior. It is our proposal that such ventral pathway damage could help distinguish clinico-anatomical correlates of motor versus conceptual apraxias.

Core to the goals of this Research Topic (“Bridging the theories of offordances and limb apraxia”), what does MSAG have to do with apraxia? We have had interest in focusing MSAG on the conceptual level, to better understand neural circuits that could be vulnerable in persons with conceptual apraxias. We have recently refined the MSAG proposal, suggesting that the dorsal parietofrontal areas encode possible “functional affordance,” where qualities of seen (or desired) actions of tools are encoded based on relevance to behaviors for achievement of an action plan (Mizelle et al., 2013). In this work, we chose tool-object pairs that were always correct/possible to fulfill an action goal, but modified the functional affordance by changing how the tool interacted with the object. When functional affordance is high (correct tool-object pairs are used correctly) parietofrontal activation is dominant. Yet, when functional affordance is low (correct tool-object pairs used incorrectly), ventral brain areas show significant activations. Thus, functional affordance may be similarly driven, at least in part, by the mechanical/physical alignment (Mizelle et al., 2013) and contextualization (Mizelle and Wheaton, 2010a,d) of tool-object pairs. This helps to underscore a common problem in conceptual apraxia, where tool selection for a task is impacted. If ventral networks are largely affected, conceptual errors can become predominant, yet the MSAG model does not stop there.

As MSAG predicts, successful fulfillment of the action goal is paramount and a certain amount of inherent flexibility exists to accomplish the goal. As we proposed in MSAG, accomplishing the goal without ending in a fault is of primary concern. MSAG proposes a preliminary framework of how both conceptual and motor “faults” may occur, which would reflect conceptual and ideomotor apraxias. While many of our studies have focused on the conceptual errors, we are continuing work on expansion into the motoric domain, and the interactions between conceptual and motor properties of action. We anticipate that such refinement will be a pivotal step in being able to better detail the neural systems involved in apraxia.

We are continuing to study how we encode and understand action goals, which is core to shaping MSAG and highly relevant to the opinion article by Ousirak. In the context of goal-based tool use, our own work suggests that affordances are complex, possibly dynamic entities. We propose that research should focus on the varied properties of affordance and how these varied properties might interact. A good place to start would be seeking to align the various proposals of affordance, and their relevance to apraxia, through collaborative research efforts.

## Conflict of interest statement

The authors declare that the research was conducted in the absence of any commercial or financial relationships that could be construed as a potential conflict of interest.

## References

[B1] Mizelle J. C. Kelly R. L. Wheaton L. A. (2013). Ventral encoding of functional affordances: a neural pathway for identifying errors in action. Brain Cogn. 82, 274–282 10.1016/j.bandc.2013.05.002 23733029

[B2] Mizelle J. C. Wheaton L. A. (2010a). Neural activation for conceptual identification of correct versus incorrect tool-object pairs. Brain Res. 1354, 100–112 10.1016/j.brainres.2010.07.059 20701898

[B3] Mizelle J. C. Wheaton L. A. (2010b). The neuroscience of storing and molding tool action concepts: how “plastic” is grounded cognition?. Front. Psychol. 1:195 10.3389/fpsyg.2010.00195 21833254 PMC3153804

[B4] Mizelle J. C. Wheaton L. A. (2010c). Testing perceptual limits of functional units: are there automatic tendencies to associate tools and objects. Neurosci. Lett. 92–96 10.1016/j.neulet.2010.11.009 21073916

[B5] Mizelle J. C. Wheaton L. A. (2010d). Why is that hammer in my coffee: a multimodal imaging investigation of contextually-based tool understanding. Front. Hum. Neurosci. 4:233 10.3389/fnhum.2010.00233 21228903 PMC3016621

[B6] Osiurak F. (2013). Apraxia of tool use is not a matter of affordances. Front. Hum. Neurosci. 7:890 10.3389/fnhum.2013.00890 24391576 PMC3868885

[B7] Pellicano A. Thill S. Ziemke T. Binkofski F. (2011). Affordances, adaptive tool use and grounded cognition. Front. Psychol. 2:53 10.3389/fpsyg.2011.00053 21716579 PMC3110812

